# Supplementary material for: Whole-genome sequencing and analysis of two azaleas, Rhododendron ripense and Rhododendron kiyosumense
Source: DNA Res. 2021 Jul 20;28(5):dsab010. doi: 10.1093/dnares/dsab010 (PMC8435550; doi:10.1093/dnares/dsab010)
Supplement: dsab010_Supplementary_Data [file dsab010_supplementary_data.zip › AzaleaGenomeSupplementaryFigures.pdf]

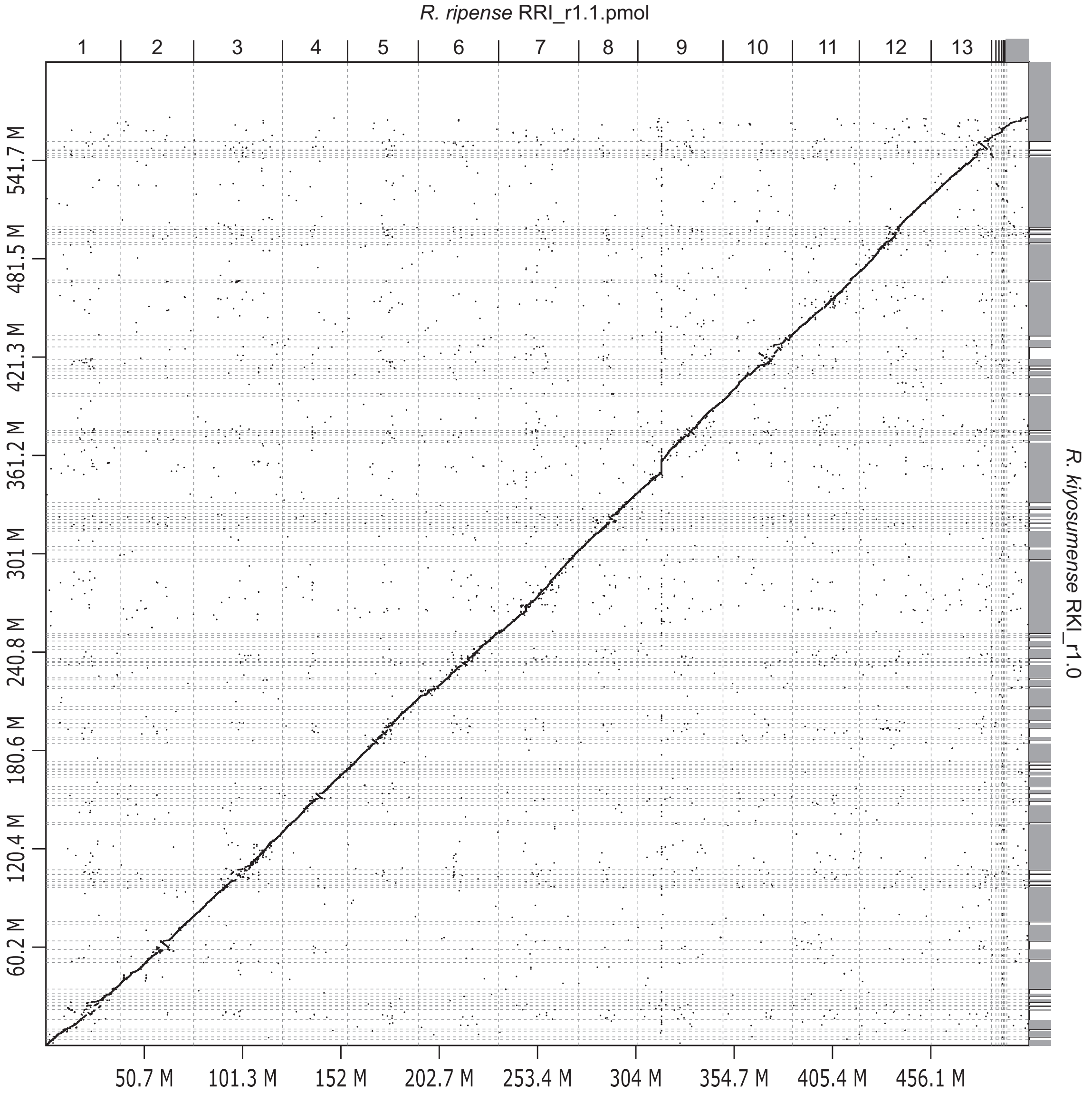

Supplementary Figure S1 Alignment of the *Rhododendron kiyosumense* genome assembly against the *Rhododendron ripense* pseudomolecule sequences.

The dot plot shows the collinearity between the genome assembly of *R. kiyosumense* (y-axis) and that of *R. ripense* (x-axis).

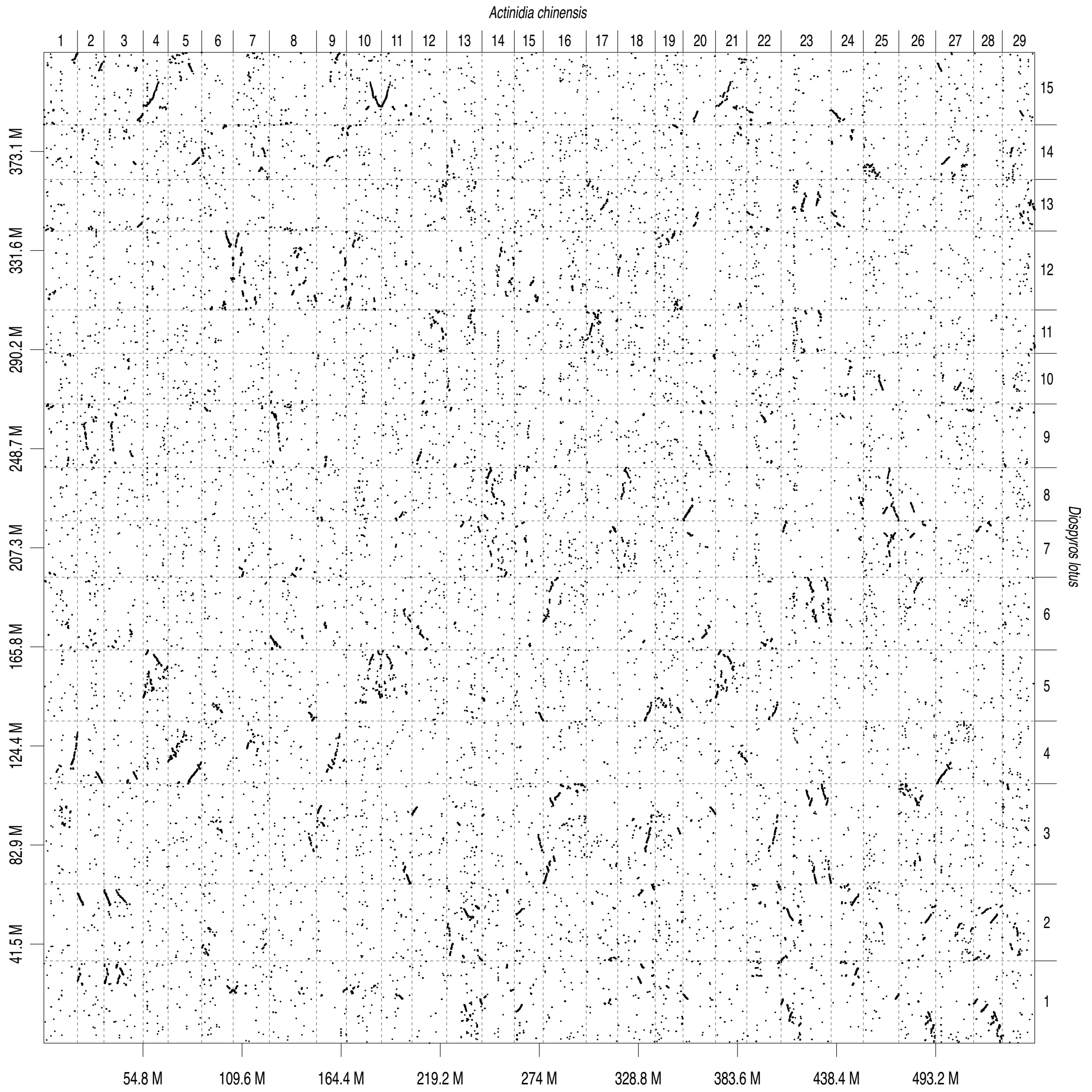

Supplementary Figure S2 Comparative analysis of the genome sequence and structure of Actinidia chinensis and Diospyros lotus.

Similarities in the genome sequence and structure are shown by dots.
